# Crystal structure of *N*-[3-(di­methyl­aza­nium­yl)prop­yl]-*N*′,*N*′,*N*′′,*N*′′-tetra­methyl-*N*-(*N*,*N*,*N*′,*N*′-tetra­methyl­form­am­id­in­ium­yl)­guanidinium dibromide hydroxide monohydrate

**DOI:** 10.1107/S2056989015024305

**Published:** 2015-12-24

**Authors:** Ioannis Tiritiris, Willi Kantlehner

**Affiliations:** a Fakultät Chemie/Organische Chemie, Hochschule Aalen, Beethovenstrasse 1, D-73430 Aalen, Germany

**Keywords:** crystal structure, bis­amidinium salt, bromide, hydroxide, hydrate, hydrogen bonds

## Abstract

The asymmetric unit of the title hydrated salt, C_15_H_37_N_6_^3+^·2Br^−^·OH^−^·H_2_O, contains one cation, three partial-occupancy bromide ions, one hydroxide ion and one water mol­ecule. Refinement of the site-occupancy factors of the three disordered bromide ions converges with occupancies 0.701 (2), 0.831 (2) and 0.456 (2) summing to approximately two bromide ions per formula unit. The structure was refined as a two-component inversion twin with volume fractions 0.109 (8):0.891 (8) for the two domains. The central C_3_N unit of the bis­amidinium ion is linked to the aliphatic propyl chain by a C—N single bond. The other two bonds in this unit have double-bond character as have the four C—N bonds to the outer NMe_2_ groups. In contrast, the three C—N bonds to the central N atom of the (di­methyl­aza­nium­yl)propyl group have single-bond character. Delocalization of the two positive charges occurs in the N/C/N and C/N/C planes, while the third positive charge is localized on the di­methyl­ammonium group. The crystal structure is stabilized by O—H⋯O, N—H⋯Br, O—H⋯Br and C—H⋯Br hydrogen bonds, forming a three-dimensional network.

## Related literature   

For the crystal structure of *N*,*N*,*N*′,*N*′-tetra­methyl­chloro­formamidinium chloride, see: Tiritiris & Kantlehner (2008 ▸); for ethyl­tri­phenyl­phospho­nium bromide dihydrate, see: Betz & Gerber (2011 ▸); for *N*-[3-(di­methyl­amino)­prop­yl]-*N*-(*N*,*N*,*N*′,*N*′-tetra­methyl-formamidinium­yl)-*N*′,*N*′,*N*′′,*N*′′-tetra­methyl­guanidinium bis­(tetra­phenyl­borate), see: Tiritiris & Kantlehner (2015 ▸). For the synthesis of *N*′′-[3-(di­methyl­amino)­prop­yl]-*N*,*N*,*N*′,*N*′-tetra­methyl­guanidine, see: Tiritiris & Kantlehner (2012 ▸).
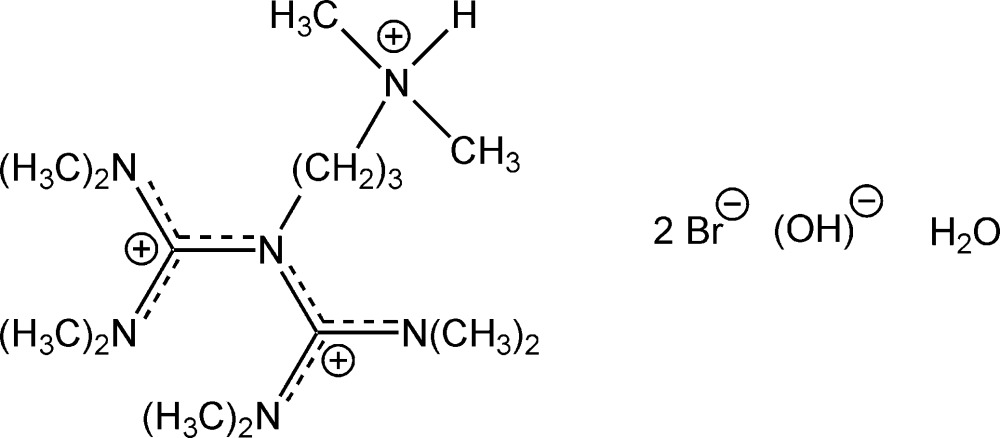


## Experimental   

### Crystal data  

C_15_H_37_N_6_^3+^·1.988Br^−^·OH^−^·H_2_O
*M* *_r_* = 495.37
Monoclinic, 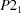

*a* = 9.1584 (6) Å
*b* = 12.2932 (7) Å
*c* = 10.6633 (6) Å
β = 97.454 (3)°
*V* = 1190.39 (12) Å^3^
*Z* = 2
Mo *K*α radiation
μ = 3.40 mm^−1^
*T* = 100 K
0.41 × 0.29 × 0.25 mm

### Data collection  

Bruker Kappa APEXII DUO diffractometer
Absorption correction: multi-scan (Blessing, 1995 ▸) *T*_min_ = 0.334, *T*_max_ = 0.481
25793 measured reflections
7244 independent reflections
6391 reflections with *I* > 2σ(*I*)
*R*_int_ = 0.033

### Refinement  

*R*[*F* ^2^ > 2σ(*F* ^2^)] = 0.031
*wR*(*F* ^2^) = 0.069
*S* = 0.99
7244 reflections
265 parameters
1 restraint
H atoms treated by a mixture of independent and constrained refinement
Δρ_max_ = 0.37 e Å^−3^
Δρ_min_ = −0.23 e Å^−3^
Absolute structure: refined as an inversion twin
Absolute structure parameter: 0.109 (8)

### 

Data collection: *APEX2* (Bruker, 2008 ▸); cell refinement: *SAINT* (Bruker, 2008 ▸); data reduction: *SAINT*; program(s) used to solve structure: *SHELXS97* (Sheldrick, 2008 ▸); program(s) used to refine structure: *SHELXL2014* (Sheldrick, 2015 ▸); molecular graphics: *DIAMOND* (Brandenburg & Putz, 2005 ▸); software used to prepare material for publication: *SHELXL2014*.

## Supplementary Material

Crystal structure: contains datablock(s) I, global. DOI: 10.1107/S2056989015024305/sj5490sup1.cif

Structure factors: contains datablock(s) I. DOI: 10.1107/S2056989015024305/sj5490Isup2.hkl

**Fig. 1.**
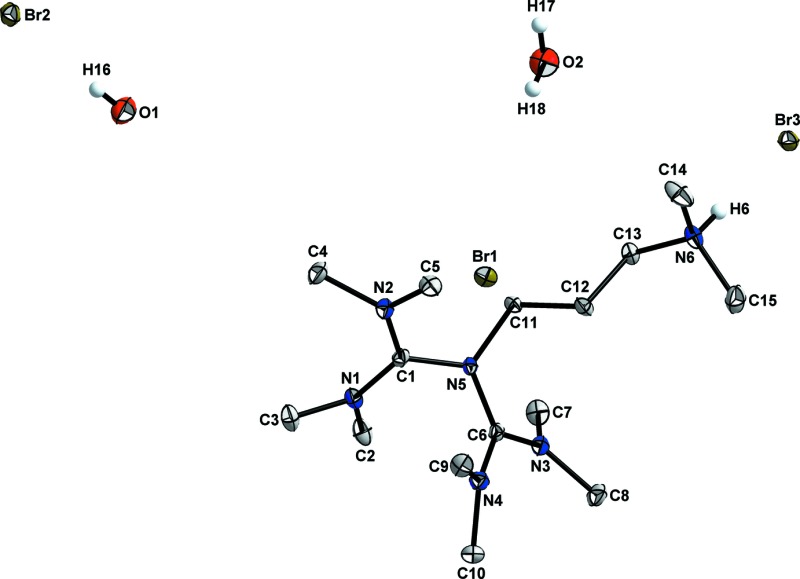
The structure of the title compound with displacement ellipsoids at the 50% probability level. All carbon-bonded hydrogen atoms are omitted for the sake of clarity.

**Fig. 2.**
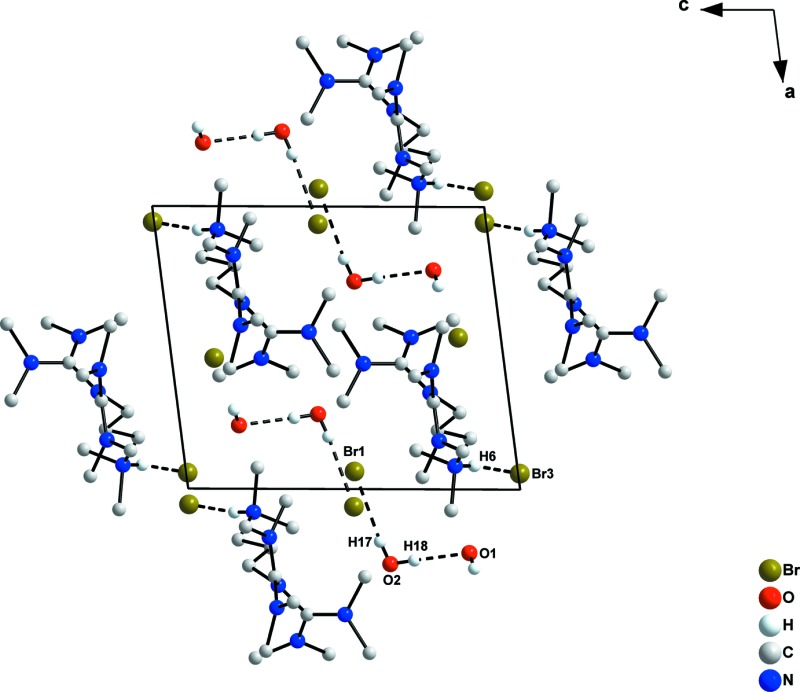
N—H···Br, O—H···Br and O—H···O hydrogen bonds (black dashed lines) in the crystal structure of the title compound (ac view).

**Fig. 3.**
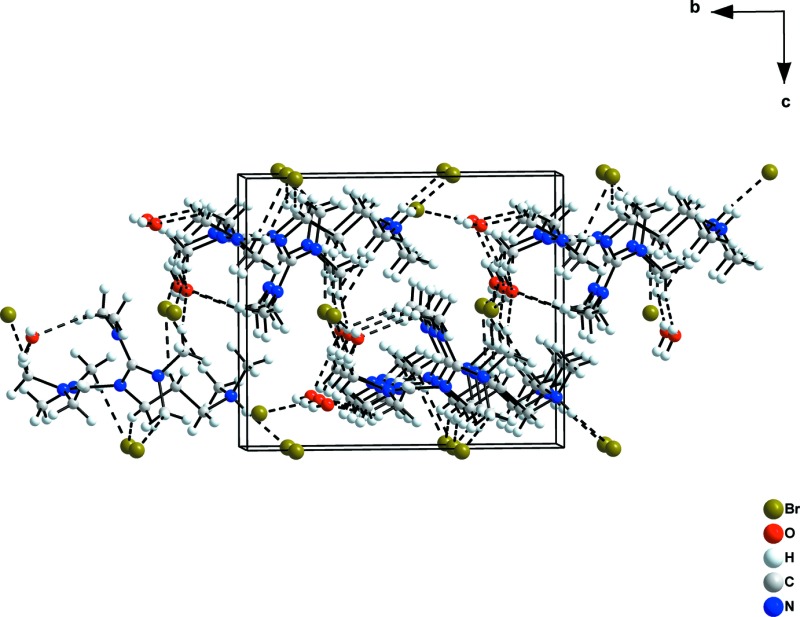
Molecular packing of the title compound (bc view). The N—H···Br, O—H···Br, O—H···O and C—H···Br hydrogen bonds are depicted by black dashed lines.

. DOI: 10.1107/S2056989015024305/sj5490fig1.tif
The structure of the title compound with displacement ellipsoids at the 50% probability level. All carbon-bonded hydrogen atoms are omitted for the sake of clarity.

ac . DOI: 10.1107/S2056989015024305/sj5490fig2.tif
N—H⋯Br, O—H⋯Br and O—H⋯O hydrogen bonds (black dashed lines) in the crystal structure of the title compound (*ac* view).

bc . DOI: 10.1107/S2056989015024305/sj5490fig3.tif
Mol­ecular packing of the title compound (*bc* view). The N—H⋯Br, O—H⋯Br, O—H⋯O and C—H⋯Br hydrogen bonds are depicted by black dashed lines.

CCDC reference: 1443022

Additional supporting information:  crystallographic information; 3D view; checkCIF report

## Figures and Tables

**Table 1:** Hydrogen-bond geometry (Å, °)

*D*—H⋯*A*	*D*—H	H⋯*A*	*D*⋯*A*	*D*—H⋯*A*
N6—H6⋯Br3^i^	0.86 (4)	2.18 (4)	3.038 (4)	172 (3)
O2—H18⋯O1^ii^	0.86 (5)	1.96 (4)	2.825 (4)	179 (3)
O2—H17⋯Br1^iii^	0.80 (5)	2.48 (4)	3.273 (4)	171 (3)
C2—H2*A*⋯Br2^iv^	0.98	2.87	3.650 (4)	137
C3—H3*A*⋯Br1^v^	0.98	2.72	3.688 (4)	170
C5—H5*C*⋯Br3^vi^	0.98	2.69	3.594 (4)	153
C7—H7*B*⋯Br3^ii^	0.98	2.67	3.498 (4)	142
C8—H8*C*⋯Br1	0.98	2.87	3.805 (4)	160
C11—H11*A*⋯Br3^vi^	0.99	2.70	3.618 (4)	154
C12—H12*A*⋯Br1^iii^	0.99	2.75	3.649 (4)	151
C14—H14*C*⋯Br1^iii^	0.98	2.78	3.743 (4)	167
C15—H15*B*⋯Br1^ii^	0.98	2.86	3.676 (4)	142

Symmetry codes: (i) 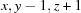; (ii) 
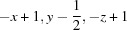
; (iii) 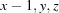; (iv) 
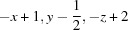
; (v) 
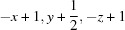
; (vi) 
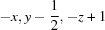
.

## supplementary crystallographic information

### S1. Comment

*N*''-[3-(dimethylamino)propyl]- *N*,*N*,*N*',*N*'-tetramethylguanidine (Tiritiris & Kantlehner, 2012) reacts with one equivalent of *N*,*N*,*N*',*N*'- tetramethylchloroformamidinium chloride (Tiritiris & Kantlehner, 2008), yielding *N*-[3-(dimethylamino)propyl]- *N*-(*N*,*N*,*N*',*N*'-tetramethyl-formamidinio)- *N*',*N*',*N*'',*N*''-tetramethylguanidinium dichloride as the product. As expected, on protonation with acid, the terminal 3-(dimethylamino)propyl group can be converted into a 3-(dimethylammonio)propyl group and a triply charged cationic species is formed. The crystal structure presented here is the first structural study of a tricationic nonasubstituted bisamidinium salt. The asymmetric unit contains one cation, three partial occupancy bromide ions, one hydroxide ion and one water molecule (Fig. 1). The sites of the disordered bromine atoms are not fully occupied, the refinement of their site occupation factors converges to Br = [Br1 + Br2 + Br3] = [0.701 (2) + 0.831 (2) + 0.456 (2) = 1.988 (2)] resulting in approximately two bromide ions per formula unit. Prominent bond parameters in the bisamidinium ion are: N5–C6 = 1.390 (3) Å, N5–C1 = 1.399 (4) Å, N5–C11 = 1.494 (4) Å, indicating the N–C single- and double-bond character of the central C_3_N unit. The C–N–C angles are 119.6 (3)°, 119.8 (2)° and 120.4 (2)°, signalling a nearly ideal trigonal-planar arangement about the central N5 nitrogen atom by the C1, C6 and C11 carbon atoms. These carbon atoms are further bound to the N1, N2, N3 and N4 nitrogen atoms and the resulting C–N bonds show double-bond character with bond lengths in the range 1.326 (4) Å to 1.335 (4) Å. The N–C–N angles range from 118.7 (3)° to 122.5 (3)°, again indicating almost ideal trigonal-planar surroundings of both carbon centres by the nitrogen atoms. The dihedral angle between the N1/C1/N2 and N3/C6/N4 planes is 70.1 (3)°. Structural data for the cation agree very well with those from the crystal structure analysis of *N*-[3-(dimethylamino)propyl]- *N*-(*N*,*N*,*N*',*N*'-tetramethyl-formamidinio)- *N*',*N*',*N*'',*N*''-tetramethylguanidinium bis(tetraphenylborate) (Tiritiris & Kantlehner, 2015). Two of the positive charges are delocalized in the N1/C1/N2, N3/C6/N4 and C1/N5/C6, planes the third positive charge is localized on the dimethylammonium group. The N–C bond lengths in the terminal ammonium group are in a range from 1.492 (4) to 1.494 (4) Å. A strong N–H···Br hydrogen bond forms between the hydrogen atom H6 of the ammonium group and one of the bromide ions (Br3) [*d*(H···Br) = 2.18 (4) Å, (Tab.1)]. O–H···O hydrogen bonds [*d*(H···O) = 1.96 (4) Å, (Table 1)] between the water molecule and the hydroxide ion and O–H···Br hydrogen bonds between the water molecule and the bromide ion [*d*(H···Br) = 2.48 (4) Å, (Table 1)] are also observed (Fig. 2). In addition, C–H···Br interactions are apparent between the bisamidimium hydrogen atoms of –N(CH_3_)_2_ and –CH_2_ groups and the bromide ions [*d*(H···Br) = 2.67 − 2.87 Å, (Tab.1)], forming a three-dimensional network (Fig. 3). Similar H···Br distances have been observed in the crystal structure of ethyltriphenylphosphonium bromide dihydrate (Betz & Gerber, 2011) for both the O–H···Br and C–H···Br hydrogen bonds.

### S2. Experimental

The title compound was prepared by treating an aqueous solution of *N*-[3-(dimethylamino)propyl]-*N*-(*N*,*N*,*N*', *N*'-tetramethyl-formamidinio)-*N*',*N*',*N*'',*N*''- tetramethylguanidinium dichloride with hydrobromic acid (48 wt.% in H_2_O). After slow evaporation of the water at ambient temperature, colorless single crystals of the title compound emerged.

### S3. Refinement

The O-bound and N-bound H atoms were located in a difference Fourier map and were refined freely [O—H = 0.75 (5) − 0.86 (5) Å; N—H = 0.86 (4) Å]. The title compound crystallizes in the non-centrosymmetric space group *P2_1_*; the crystal was refined as a 2-component inversion twin using the matrix [−1 0 0 0 − 1 0 0 0 − 1] with a volume fraction of 0.109 (8):0.891 (8) for the two domains. The positions of the bromide ions were not fully occupied and their site occupancy factors were refined and converged to Br1 = 0.701 (2), Br2 = 0.831 (2), Br3 = 0.456 (2). The hydrogen atoms of the methyl groups were allowed to rotate with a fixed angle around the C–N bond to best fit the experimental electron density, with *U*_iso_(H) set to 1.5*U*_eq_(C) and d(C—H) = 0.98 Å. The remaining H atoms were placed in calculated positions with d(C—H) = 0.99 Å (H atoms in CH_2_ groups) and were refined using a riding model, with *U*_iso_(H) set to 1.2 *U*_eq_(C).

### Crystal data

**Table:**

C_15_H_37_N_6_^3+^·1.988Br^−^·OH^−^·H_2_O	*F*(000) = 515.2
*M**_r_* = 495.37	*D*_x_ = 1.382 Mg m^−^^3^
Monoclinic, *P*2_1_	Mo *K*α radiation, λ = 0.71073 Å
*a* = 9.1584 (6) Å	Cell parameters from 25793 reflections
*b* = 12.2932 (7) Å	θ = 1.9–30.5°
*c* = 10.6633 (6) Å	µ = 3.40 mm^−^^1^
β = 97.454 (3)°	*T* = 100 K
*V* = 1190.39 (12) Å^3^	Prism, colorless
*Z* = 2	0.41 × 0.29 × 0.25 mm

### Data collection

**Table:**

Bruker Kappa APEXII DUO diffractometer	7244 independent reflections
Radiation source: fine-focus sealed tube	6391 reflections with *I* > 2σ(*I*)
Triumph monochromator	*R*_int_ = 0.033
φ scans, and ω scans	θ_max_ = 30.5°, θ_min_ = 1.9°
Absorption correction: multi-scan (Blessing, 1995)	*h* = −13→12
*T*_min_ = 0.334, *T*_max_ = 0.481	*k* = −17→17
25793 measured reflections	*l* = −15→15

### Refinement

**Table:**

Refinement on *F*^2^	Secondary atom site location: difference Fourier map
Least-squares matrix: full	Hydrogen site location: inferred from neighbouring sites
*R*[*F*^2^ > 2σ(*F*^2^)] = 0.031	H atoms treated by a mixture of independent and constrained refinement
*wR*(*F*^2^) = 0.069	*w* = 1/[σ^2^(*F*_o_^2^) + (0.0344*P*)^2^] where *P* = (*F*_o_^2^ + 2*F*_c_^2^)/3
*S* = 0.99	(Δ/σ)_max_ < 0.001
7244 reflections	Δρ_max_ = 0.37 e Å^−^^3^
265 parameters	Δρ_min_ = −0.23 e Å^−^^3^
1 restraint	Absolute structure: refined as an inversion twin
Primary atom site location: structure-invariant direct methods	Absolute structure parameter: 0.109 (8)

### Special details

**Table:**

Geometry. All e.s.d.'s (except the e.s.d. in the dihedral angle between two l.s. planes) are estimated using the full covariance matrix. The cell e.s.d.'s are taken into account individually in the estimation of e.s.d.'s in distances, angles and torsion angles; correlations between e.s.d.'s in cell parameters are only used when they are defined by crystal symmetry. An approximate (isotropic) treatment of cell e.s.d.'s is used for estimating e.s.d.'s involving l.s. planes.
Refinement. Refinement of *F*^2^ against ALL reflections. The weighted *R*-factor *wR* and goodness of fit *S* are based on *F*^2^, conventional *R*-factors *R* are based on *F*, with *F* set to zero for negative *F*^2^. The threshold expression of *F*^2^ > σ(*F*^2^) is used only for calculating *R*-factors(gt) *etc*. and is not relevant to the choice of reflections for refinement. *R*-factors based on *F*^2^ are statistically about twice as large as those based on *F*, and *R*- factors based on ALL data will be even larger. The crystal was refined as a 2-component inversion twin.

### Fractional atomic coordinates and isotropic or equivalent isotropic displacement parameters (Å^2^)

**Table:**

	*x*	*y*	*z*	*U*_iso_*/*U*_eq_	Occ. (<1)
Br1	0.93762 (4)	0.22807 (3)	0.49250 (4)	0.01601 (14)	0.701 (2)
Br2	0.53705 (4)	0.94972 (3)	0.87115 (3)	0.01916 (12)	0.831 (2)
Br3	0.05637 (7)	0.84749 (5)	0.00432 (6)	0.0171 (2)	0.456 (2)
O1	0.7750 (3)	0.7662 (2)	0.8224 (3)	0.0300 (6)	
H16	0.720 (5)	0.809 (4)	0.831 (4)	0.033 (13)*	
C1	0.3100 (3)	0.4939 (2)	0.7726 (2)	0.0129 (6)	
N1	0.4205 (3)	0.5645 (2)	0.7784 (2)	0.0150 (5)	
N2	0.1715 (3)	0.5246 (2)	0.7743 (2)	0.0158 (5)	
C2	0.5739 (3)	0.5375 (3)	0.8281 (3)	0.0196 (6)	
H2A	0.5747	0.4800	0.8919	0.029*	
H2B	0.6231	0.6023	0.8668	0.029*	
H2C	0.6258	0.5122	0.7588	0.029*	
C3	0.4007 (4)	0.6751 (2)	0.7265 (3)	0.0205 (6)	
H3A	0.3067	0.6796	0.6712	0.031*	
H3B	0.4814	0.6922	0.6777	0.031*	
H3C	0.4009	0.7274	0.7959	0.031*	
C4	0.1345 (4)	0.6237 (3)	0.8414 (3)	0.0234 (7)	
H4A	0.2218	0.6491	0.8965	0.035*	
H4B	0.0558	0.6074	0.8926	0.035*	
H4C	0.1013	0.6804	0.7797	0.035*	
C5	0.0433 (3)	0.4656 (3)	0.7104 (3)	0.0182 (6)	
H5A	0.0770	0.4034	0.6643	0.027*	
H5B	−0.0150	0.5144	0.6508	0.027*	
H5C	−0.0175	0.4396	0.7734	0.027*	
C6	0.4445 (3)	0.3476 (2)	0.6899 (3)	0.0118 (5)	
N3	0.5375 (3)	0.26813 (19)	0.7292 (2)	0.0141 (5)	
N4	0.4440 (3)	0.3922 (2)	0.5757 (2)	0.0146 (5)	
C7	0.5991 (3)	0.2503 (3)	0.8627 (3)	0.0182 (6)	
H7A	0.5651	0.3081	0.9152	0.027*	
H7B	0.7069	0.2514	0.8704	0.027*	
H7C	0.5661	0.1796	0.8909	0.027*	
C8	0.5846 (4)	0.1889 (2)	0.6400 (3)	0.0194 (6)	
H8A	0.5175	0.1916	0.5606	0.029*	
H8B	0.5832	0.1157	0.6763	0.029*	
H8C	0.6848	0.2064	0.6234	0.029*	
C9	0.3103 (3)	0.4361 (3)	0.5014 (3)	0.0187 (6)	
H9A	0.2244	0.3945	0.5200	0.028*	
H9B	0.3193	0.4304	0.4110	0.028*	
H9C	0.2983	0.5126	0.5236	0.028*	
C10	0.5804 (3)	0.4063 (3)	0.5171 (3)	0.0203 (6)	
H10A	0.6647	0.3800	0.5752	0.031*	
H10B	0.5941	0.4835	0.4990	0.031*	
H10C	0.5729	0.3646	0.4381	0.031*	
N5	0.3413 (3)	0.38281 (19)	0.7657 (2)	0.0113 (5)	
C11	0.2721 (3)	0.3039 (2)	0.8469 (3)	0.0126 (5)	
H11A	0.1924	0.3411	0.8846	0.015*	
H11B	0.3469	0.2800	0.9169	0.015*	
C12	0.2085 (3)	0.2040 (2)	0.7739 (3)	0.0149 (6)	
H12A	0.1152	0.2233	0.7209	0.018*	
H12B	0.2786	0.1771	0.7178	0.018*	
C13	0.1805 (3)	0.1162 (2)	0.8688 (3)	0.0141 (6)	
H13A	0.2761	0.0846	0.9054	0.017*	
H13B	0.1352	0.1501	0.9385	0.017*	
N6	0.0827 (3)	0.0267 (2)	0.8125 (2)	0.0159 (5)	
H6	0.084 (4)	−0.022 (3)	0.870 (4)	0.032 (11)*	
C14	−0.0748 (3)	0.0601 (3)	0.7838 (3)	0.0254 (7)	
H14A	−0.1061	0.0957	0.8581	0.038*	
H14B	−0.1359	−0.0043	0.7621	0.038*	
H14C	−0.0857	0.1109	0.7123	0.038*	
C15	0.1372 (4)	−0.0259 (3)	0.7013 (3)	0.0246 (7)	
H15A	0.1264	0.0247	0.6297	0.037*	
H15B	0.0797	−0.0918	0.6783	0.037*	
H15C	0.2412	−0.0452	0.7229	0.037*	
O2	0.2652 (3)	0.1697 (2)	0.4200 (3)	0.0299 (6)	
H17	0.185 (6)	0.177 (4)	0.442 (4)	0.045 (14)*	
H18	0.252 (5)	0.200 (4)	0.346 (5)	0.049 (14)*	

### Atomic displacement parameters (Å^2^)

**Table:**

	*U*^11^	*U*^22^	*U*^33^	*U*^12^	*U*^13^	*U*^23^
Br1	0.0157 (2)	0.0191 (2)	0.0127 (2)	−0.00070 (16)	−0.00014 (14)	0.00164 (16)
Br2	0.01885 (18)	0.01493 (17)	0.02373 (19)	0.00227 (15)	0.00287 (13)	0.00258 (15)
Br3	0.0172 (4)	0.0156 (4)	0.0191 (4)	0.0025 (2)	0.0050 (3)	0.0041 (2)
O1	0.0258 (13)	0.0279 (14)	0.0368 (15)	−0.0011 (11)	0.0059 (11)	−0.0102 (11)
C1	0.0176 (14)	0.0119 (13)	0.0090 (12)	0.0003 (11)	0.0016 (10)	−0.0012 (10)
N1	0.0173 (12)	0.0124 (12)	0.0149 (12)	−0.0028 (10)	0.0005 (9)	0.0002 (10)
N2	0.0184 (13)	0.0124 (12)	0.0173 (12)	0.0015 (10)	0.0043 (10)	0.0000 (10)
C2	0.0197 (15)	0.0169 (15)	0.0203 (15)	−0.0047 (12)	−0.0048 (12)	0.0008 (12)
C3	0.0268 (17)	0.0121 (14)	0.0220 (15)	−0.0028 (12)	0.0005 (13)	0.0022 (12)
C4	0.0311 (18)	0.0163 (15)	0.0245 (16)	0.0071 (14)	0.0105 (14)	−0.0020 (13)
C5	0.0131 (13)	0.0179 (17)	0.0234 (15)	0.0016 (11)	0.0017 (11)	0.0031 (12)
C6	0.0112 (12)	0.0109 (13)	0.0132 (13)	−0.0017 (10)	0.0013 (10)	−0.0036 (10)
N3	0.0135 (12)	0.0125 (12)	0.0161 (12)	0.0005 (9)	0.0015 (9)	−0.0014 (9)
N4	0.0159 (12)	0.0147 (12)	0.0133 (11)	0.0010 (10)	0.0029 (9)	0.0010 (10)
C7	0.0149 (14)	0.0190 (16)	0.0188 (14)	0.0017 (11)	−0.0044 (11)	0.0018 (12)
C8	0.0216 (16)	0.0151 (14)	0.0222 (15)	0.0066 (12)	0.0057 (12)	0.0000 (12)
C9	0.0231 (15)	0.0188 (16)	0.0137 (13)	0.0035 (13)	0.0000 (11)	0.0026 (12)
C10	0.0224 (16)	0.0199 (15)	0.0210 (15)	−0.0015 (12)	0.0112 (13)	−0.0003 (12)
N5	0.0123 (11)	0.0099 (11)	0.0118 (11)	−0.0025 (9)	0.0018 (9)	−0.0016 (9)
C11	0.0149 (13)	0.0109 (13)	0.0124 (12)	−0.0010 (10)	0.0032 (10)	0.0016 (10)
C12	0.0169 (14)	0.0139 (14)	0.0137 (13)	−0.0055 (11)	0.0016 (11)	0.0007 (10)
C13	0.0163 (14)	0.0116 (13)	0.0138 (13)	−0.0018 (11)	−0.0007 (11)	0.0010 (11)
N6	0.0167 (12)	0.0121 (12)	0.0182 (12)	−0.0019 (10)	−0.0010 (10)	0.0032 (10)
C14	0.0135 (14)	0.0285 (18)	0.0328 (19)	−0.0045 (13)	−0.0023 (13)	0.0104 (15)
C15	0.0351 (19)	0.0179 (18)	0.0201 (15)	−0.0014 (13)	0.0008 (14)	−0.0047 (12)
O2	0.0250 (14)	0.0323 (14)	0.0315 (14)	0.0052 (11)	−0.0001 (11)	0.0046 (12)

### Geometric parameters (Å, º)

**Table:**

O1—H16	0.75 (5)	C8—H8A	0.9800
C1—N2	1.326 (4)	C8—H8B	0.9800
C1—N1	1.329 (4)	C8—H8C	0.9800
C1—N5	1.399 (4)	C9—H9A	0.9800
N1—C3	1.471 (4)	C9—H9B	0.9800
N1—C2	1.474 (4)	C9—H9C	0.9800
N2—C5	1.470 (4)	C10—H10A	0.9800
N2—C4	1.474 (4)	C10—H10B	0.9800
C2—H2A	0.9800	C10—H10C	0.9800
C2—H2B	0.9800	N5—C11	1.494 (4)
C2—H2C	0.9800	C11—C12	1.528 (4)
C3—H3A	0.9800	C11—H11A	0.9900
C3—H3B	0.9800	C11—H11B	0.9900
C3—H3C	0.9800	C12—C13	1.523 (4)
C4—H4A	0.9800	C12—H12A	0.9900
C4—H4B	0.9800	C12—H12B	0.9900
C4—H4C	0.9800	C13—N6	1.494 (4)
C5—H5A	0.9800	C13—H13A	0.9900
C5—H5B	0.9800	C13—H13B	0.9900
C5—H5C	0.9800	N6—C15	1.492 (4)
C6—N3	1.328 (4)	N6—C14	1.493 (4)
C6—N4	1.335 (4)	N6—H6	0.86 (4)
C6—N5	1.390 (3)	C14—H14A	0.9800
N3—C8	1.465 (4)	C14—H14B	0.9800
N3—C7	1.478 (4)	C14—H14C	0.9800
N4—C9	1.472 (4)	C15—H15A	0.9800
N4—C10	1.477 (4)	C15—H15B	0.9800
C7—H7A	0.9800	C15—H15C	0.9800
C7—H7B	0.9800	O2—H17	0.80 (5)
C7—H7C	0.9800	O2—H18	0.86 (5)
			
N2—C1—N1	122.5 (3)	H8B—C8—H8C	109.5
N2—C1—N5	118.8 (3)	N4—C9—H9A	109.5
N1—C1—N5	118.7 (3)	N4—C9—H9B	109.5
C1—N1—C3	122.1 (3)	H9A—C9—H9B	109.5
C1—N1—C2	123.6 (2)	N4—C9—H9C	109.5
C3—N1—C2	114.1 (2)	H9A—C9—H9C	109.5
C1—N2—C5	124.1 (2)	H9B—C9—H9C	109.5
C1—N2—C4	121.5 (3)	N4—C10—H10A	109.5
C5—N2—C4	114.4 (2)	N4—C10—H10B	109.5
N1—C2—H2A	109.5	H10A—C10—H10B	109.5
N1—C2—H2B	109.5	N4—C10—H10C	109.5
H2A—C2—H2B	109.5	H10A—C10—H10C	109.5
N1—C2—H2C	109.5	H10B—C10—H10C	109.5
H2A—C2—H2C	109.5	C6—N5—C1	119.6 (2)
H2B—C2—H2C	109.5	C6—N5—C11	120.4 (2)
N1—C3—H3A	109.5	C1—N5—C11	119.8 (2)
N1—C3—H3B	109.5	N5—C11—C12	112.9 (2)
H3A—C3—H3B	109.5	N5—C11—H11A	109.0
N1—C3—H3C	109.5	C12—C11—H11A	109.0
H3A—C3—H3C	109.5	N5—C11—H11B	109.0
H3B—C3—H3C	109.5	C12—C11—H11B	109.0
N2—C4—H4A	109.5	H11A—C11—H11B	107.8
N2—C4—H4B	109.5	C13—C12—C11	108.5 (2)
H4A—C4—H4B	109.5	C13—C12—H12A	110.0
N2—C4—H4C	109.5	C11—C12—H12A	110.0
H4A—C4—H4C	109.5	C13—C12—H12B	110.0
H4B—C4—H4C	109.5	C11—C12—H12B	110.0
N2—C5—H5A	109.5	H12A—C12—H12B	108.4
N2—C5—H5B	109.5	N6—C13—C12	113.5 (2)
H5A—C5—H5B	109.5	N6—C13—H13A	108.9
N2—C5—H5C	109.5	C12—C13—H13A	108.9
H5A—C5—H5C	109.5	N6—C13—H13B	108.9
H5B—C5—H5C	109.5	C12—C13—H13B	108.9
N3—C6—N4	121.1 (3)	H13A—C13—H13B	107.7
N3—C6—N5	120.1 (3)	C15—N6—C14	111.7 (3)
N4—C6—N5	118.7 (3)	C15—N6—C13	113.2 (2)
C6—N3—C8	121.0 (2)	C14—N6—C13	113.1 (2)
C6—N3—C7	124.2 (2)	C15—N6—H6	107 (3)
C8—N3—C7	114.7 (2)	C14—N6—H6	105 (3)
C6—N4—C9	123.1 (2)	C13—N6—H6	106 (3)
C6—N4—C10	122.1 (2)	N6—C14—H14A	109.5
C9—N4—C10	114.8 (2)	N6—C14—H14B	109.5
N3—C7—H7A	109.5	H14A—C14—H14B	109.5
N3—C7—H7B	109.5	N6—C14—H14C	109.5
H7A—C7—H7B	109.5	H14A—C14—H14C	109.5
N3—C7—H7C	109.5	H14B—C14—H14C	109.5
H7A—C7—H7C	109.5	N6—C15—H15A	109.5
H7B—C7—H7C	109.5	N6—C15—H15B	109.5
N3—C8—H8A	109.5	H15A—C15—H15B	109.5
N3—C8—H8B	109.5	N6—C15—H15C	109.5
H8A—C8—H8B	109.5	H15A—C15—H15C	109.5
N3—C8—H8C	109.5	H15B—C15—H15C	109.5
H8A—C8—H8C	109.5	H17—O2—H18	101 (4)
			
N2—C1—N1—C3	−30.6 (4)	N5—C6—N4—C10	148.0 (3)
N5—C1—N1—C3	150.0 (3)	N3—C6—N5—C1	140.6 (3)
N2—C1—N1—C2	153.9 (3)	N4—C6—N5—C1	−41.9 (4)
N5—C1—N1—C2	−25.5 (4)	N3—C6—N5—C11	−34.9 (4)
N1—C1—N2—C5	149.2 (3)	N4—C6—N5—C11	142.5 (3)
N5—C1—N2—C5	−31.4 (4)	N2—C1—N5—C6	139.8 (3)
N1—C1—N2—C4	−30.2 (4)	N1—C1—N5—C6	−40.8 (4)
N5—C1—N2—C4	149.2 (3)	N2—C1—N5—C11	−44.6 (4)
N4—C6—N3—C8	−32.5 (4)	N1—C1—N5—C11	134.8 (3)
N5—C6—N3—C8	144.8 (3)	C6—N5—C11—C12	−51.0 (3)
N4—C6—N3—C7	148.6 (3)	C1—N5—C11—C12	133.5 (3)
N5—C6—N3—C7	−34.0 (4)	N5—C11—C12—C13	164.3 (2)
N3—C6—N4—C9	148.6 (3)	C11—C12—C13—N6	164.6 (2)
N5—C6—N4—C9	−28.8 (4)	C12—C13—N6—C15	55.4 (3)
N3—C6—N4—C10	−34.6 (4)	C12—C13—N6—C14	−73.0 (3)

### Hydrogen-bond geometry (Å, º)

**Table:**

*D*—H···*A*	*D*—H	H···*A*	*D*···*A*	*D*—H···*A*
N6—H6···Br3^i^	0.86 (4)	2.18 (4)	3.038 (4)	172 (3)
O2—H18···O1^ii^	0.86 (5)	1.96 (4)	2.825 (4)	179 (3)
O2—H17···Br1^iii^	0.80 (5)	2.48 (4)	3.273 (4)	171 (3)
C2—H2*A*···Br2^iv^	0.98	2.87	3.650 (4)	137
C3—H3*A*···Br1^v^	0.98	2.72	3.688 (4)	170
C5—H5*C*···Br3^vi^	0.98	2.69	3.594 (4)	153
C7—H7*B*···Br3^ii^	0.98	2.67	3.498 (4)	142
C8—H8*C*···Br1	0.98	2.87	3.805 (4)	160
C11—H11*A*···Br3^vi^	0.99	2.70	3.618 (4)	154
C12—H12*A*···Br1^iii^	0.99	2.75	3.649 (4)	151
C14—H14*C*···Br1^iii^	0.98	2.78	3.743 (4)	167
C15—H15*B*···Br1^ii^	0.98	2.86	3.676 (4)	142

Symmetry codes: (i) *x*, *y*−1, *z*+1; (ii) −*x*+1, *y*−1/2, −*z*+1; (iii) *x*−1, *y*, *z*; (iv) −*x*+1, *y*−1/2, −*z*+2; (v) −*x*+1, *y*+1/2, −*z*+1; (vi) −*x*, *y*−1/2, −*z*+1.
