# Supplementary figures and images for: Crystal structure of N-[3-(di­methyl­aza­nium­yl)prop­yl]-N′,N′,N′′,N′′-tetra­methyl-N-(N,N,N′,N′-tetra­methyl­form­am­id­in­ium­yl)­guanidinium dibromide hydroxide monohydrate
Source: Acta Crystallogr E Crystallogr Commun. 2015 Dec 24;71(Pt 12):o1078–9. doi: 10.1107/S2056989015024305 (PMC4719988; doi:10.1107/S2056989015024305)

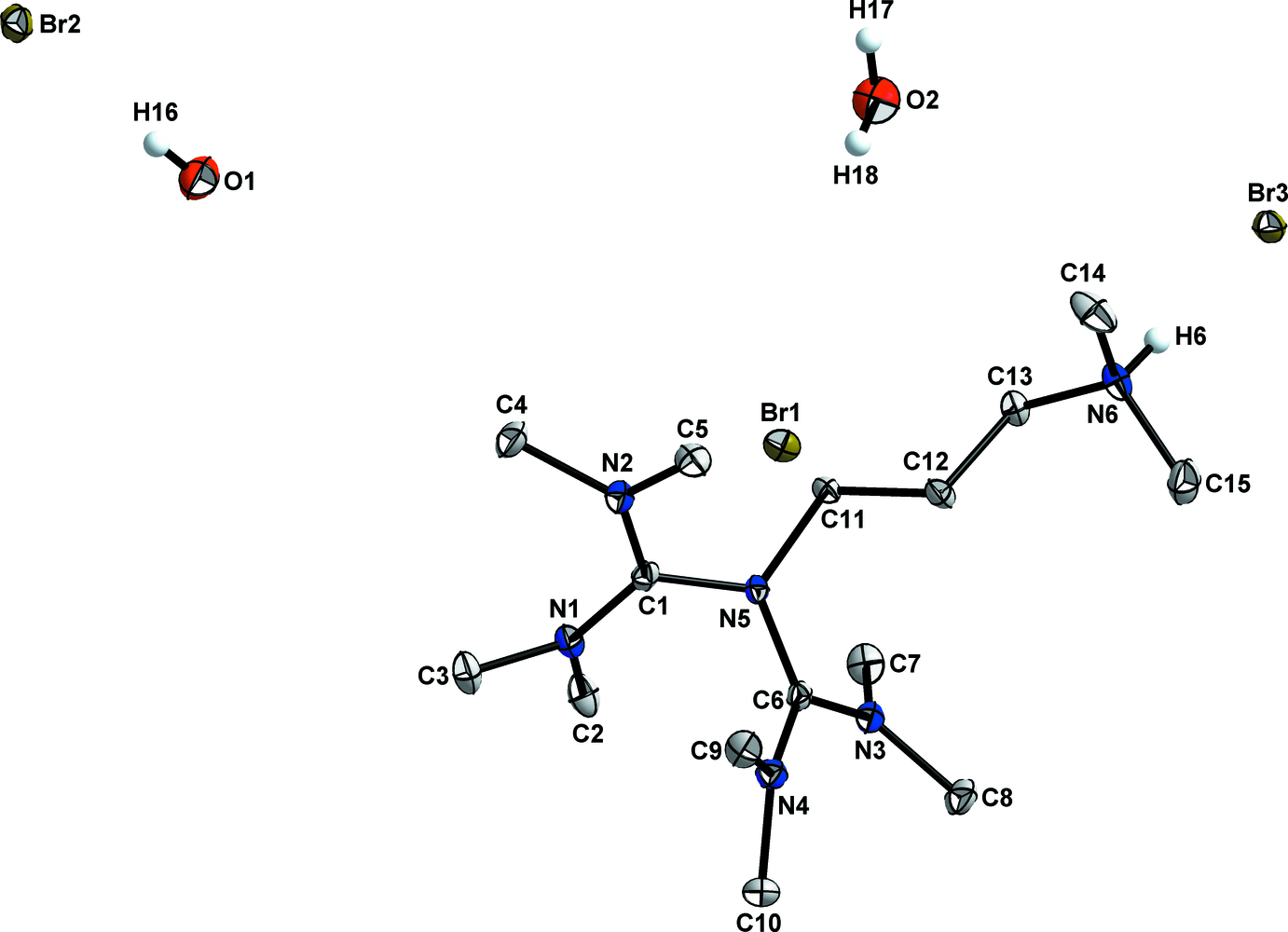

Supplement: Supplementary file 3 [file e-71-o1078-fig1.tif]

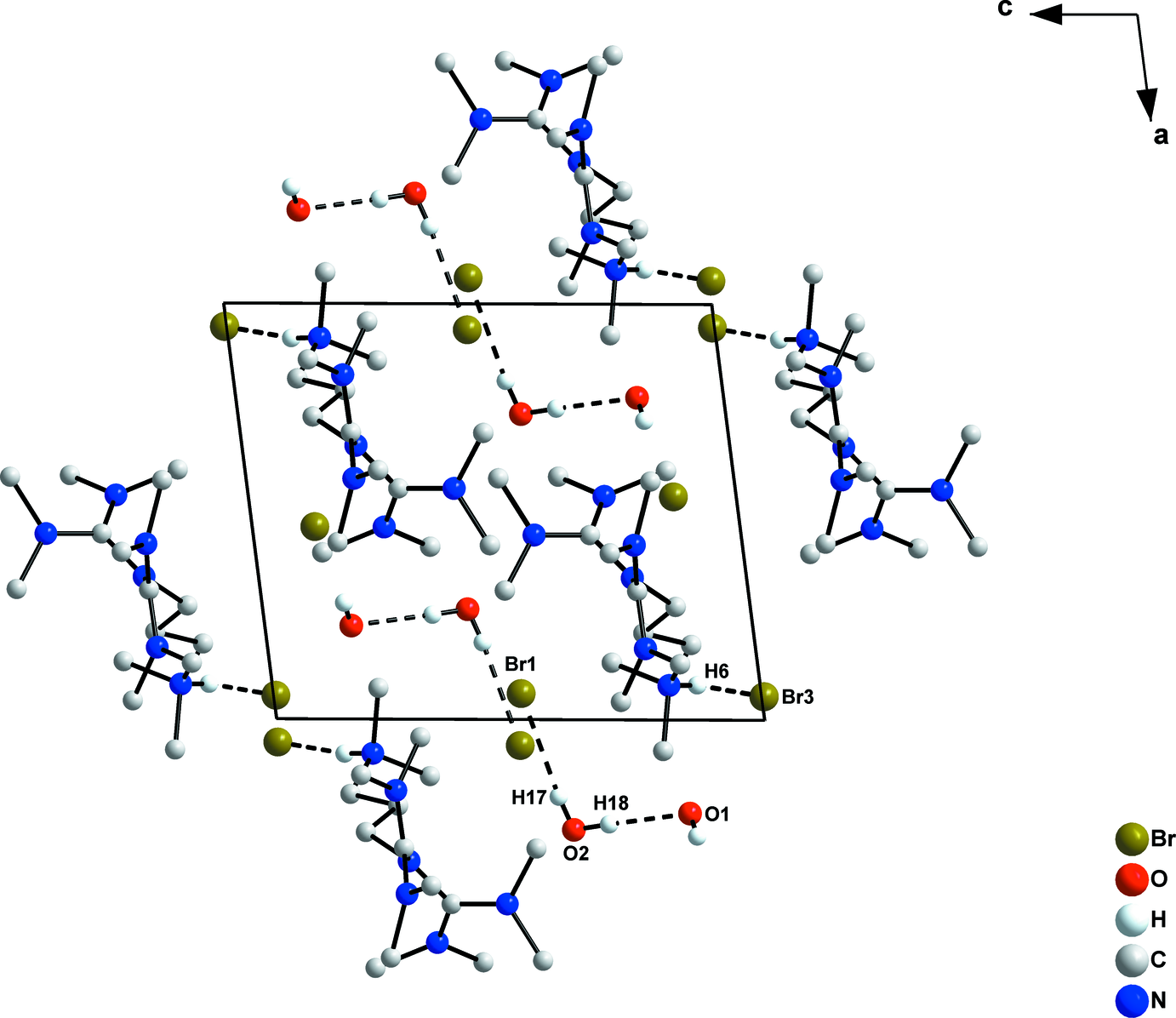

Supplement: Supplementary file 4 [file e-71-o1078-fig2.tif]

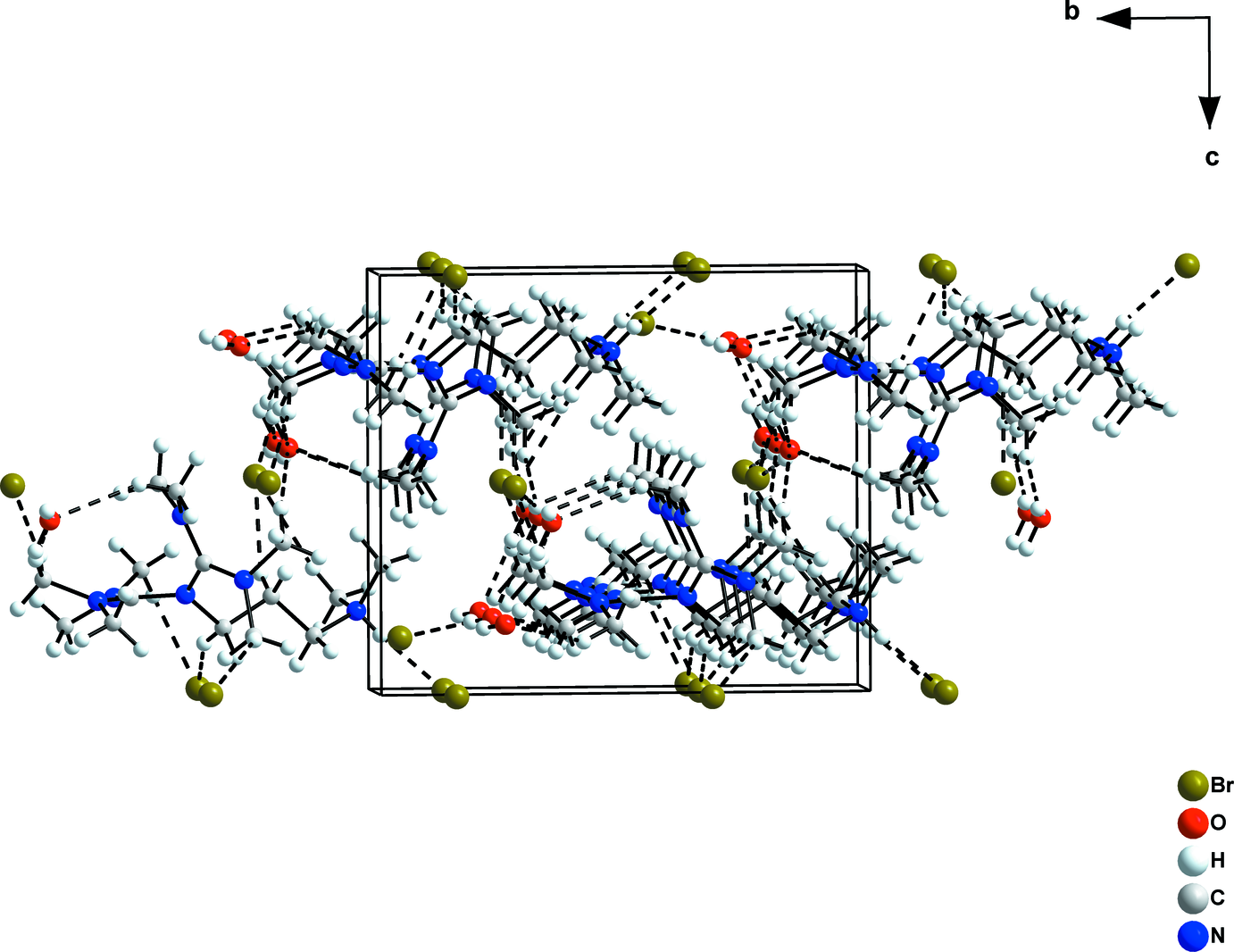

Supplement: Supplementary file 5 [file e-71-o1078-fig3.tif]
